# Generating Evidence From Contextual Clinical Research in Low- to Middle Income Countries: A Roadmap Based on Theory of Change

**DOI:** 10.3389/fped.2021.764239

**Published:** 2021-12-09

**Authors:** Babar S. Hasan, Muneera A. Rasheed, Asra Wahid, Raman Krishna Kumar, Liesl Zuhlke

**Affiliations:** ^1^Department of Paediatrics and Child Health, Aga Khan University, Karachi, Pakistan; ^2^Centre for International Health, Department of Global Public Health and Primary Care, University of Bergen, Bergen, Norway; ^3^Amrita Institute of Medical Sciences and Research Centre, Kochi, India; ^4^Division of Pediatric Cardiology, Department of Pediatrics, Red Cross Children's Hospital, University of Cape Town, Cape Town, South Africa; ^5^Division of Cardiology, Department of Medicine, Groote Schuur Hospital, University of Cape Town, Cape Town, South Africa

**Keywords:** complex care, contextual clinical research, global health, global health inequity, research disparity, theory of change

## Abstract

Along with inadequate access to high-quality care, competing health priorities, fragile health systems, and conflicts, there is an associated delay in evidence generation and research from LMICs. Lack of basic epidemiologic understanding of the disease burden in these regions poses a significant knowledge gap as solutions can only be developed and sustained if the scope of the problem is accurately defined. Congenital heart disease (CHD), for example, is the most common birth defect in children. The prevalence of CHD from 1990 to 2017 has progressively increased by 18.7% and more than 90% of children with CHD are born in Low and Middle-Income Countries (LMICs). If diagnosed and managed in a timely manner, as in high-income countries (HICs), most children lead a healthy life and achieve adulthood. However, children with CHD in LMICs have limited care available with subsequent impact on survival. The large disparity in global health research focus on this complex disease makes it a solid paradigm to shape the debate. Despite many challenges, an essential aspect of improving research in LMICs is the realization and ownership of the problem around paucity of local evidence by patients, health care providers, academic centers, and governments in these countries. We have created a theory of change model to address these challenges at a micro- (individual patient or physician or institutions delivering health care) and a macro- (government and health ministries) level, presenting suggested solutions for these complex problems. All stakeholders in the society, from government bodies, health ministries, and systems, to frontline healthcare workers and patients, need to be invested in addressing the local health problems and significantly increase data to define and improve the gaps in care in LMICs. Moreover, interventions can be designed for a more collaborative and effective HIC-LMIC and LMIC-LMIC partnership to increase resources, capacity building, and representation for long-term productivity.

## Introduction

### Why Do We Need Contextually Relevant Data From LMICs?

Low- and middle-income countries (LMICs) encompass 92% of the global disease burden (1), and yet are severely lacking in resources to manage complex care. The disparity of disease burden to resources available is daunting, i.e., HIC has over 100 times more cardiac surgeons than LMIC, and while there are more than 4,000 cardiac centers worldwide, only one center per 10 million population exists in LMIC (2). Despite these differences, there is a lack of basic epidemiologic understanding of the disease burden in these regions. A large knowledge gap is a hindrance toward identifying appropriate solutions to multifaceted healthcare challenges. This stark contrast in disease burden vs. research disparity has important implications toward complex diseases as they remain low on healthcare improvement agendas (3). We use the example of pediatric heart disease to highlight this disparity, the challenges associated with contextual evidence generation and propose some solutions to overcome these barriers.

Congenital heart disease (CHD) is the most common birth defect in children. The prevalence of CHD from 1990 to 2017 has progressively increased by 18.7% (4), and more than 90% of children with CHD are born in LMICs (5). The spectrum of CHD ranges from simple defects managed through conservative observation vs. complex conditions requiring surgical or catheter based intervention for survival. If managed in a timely manner, as in high-income countries (HICs), the majority of children lead a healthy life (5). However, children with CHD in LMICs have limited care available (6). According to Global burden of Disease (GBD) 2017, there are 261,247 deaths due to CHD annually with more than 21 million years of life (YLL) and a similarly high number of disability-adjusted life years (DALYs) lost (4). Since 1990 CHD mortality for infants has declined by only 6% in the low sociodemographic index (SDI) subgroup compared with a decline of more than 50% in the middle to high SDI subgroups (4). Additional to the disease burden, patients in LMICs have a distinct disease spectrum, often present late and with serious comorbidities (e.g., malnutrition, blood stream and lung infections) or complications (7). Inadequate quality of health care, poor infrastructure, cost of care, dearth of equipment, expertise and skills, are some of the problems unique to LMICs adding to the complexity of managing these patients (8). Due to these factors there is significantly higher pre-operative and operative mortality among these patients in LMICs when compared with HICs (9). These patients also have more pre- and post-operative morbidities significantly affecting their outcomes. More than half of pre-operative patients with CHD in LMICs had severe malnutrition (10). Compared to HIC, major infections contributed toward increased post-operative mortality and morbidity (increased ventilation time and ICU stays) in LMICs (11).

The scenario is not much different for acquired heart diseases. There has been a marked decline in the prevalence of rheumatic heart disease (RHD) in developed countries (12). However, in LMICs, RHD continues to pose a significant public health issue. A meta-analysis conducted in 2019 reported an RHD prevalence of 8.2–31.0 per 1,000 in low income countries and 5.5–13.5 per 1,000 in LMICs (13). In 2015, just five countries (India, China, Pakistan, Indonesia, and the Democratic Republic of the Congo) accounted for 73% of the global cases of RHD. Like CHD, patients with RHD also present with significantly advanced disease. Most RHD patients usually present with moderate-to-severe valvular heart disease associated with pulmonary hypertension and up to a quarter of patients present with left ventricular dysfunction, reflecting delayed referral patterns to tertiary care centers (14).

Due to the unique disease spectrum of patients with heart disease in LMICs, management needs to be tailored and demands a thoughtful and cautious approach. Late presenting left to right CHD shunt lesions are rarely seen in HICs, while it is a common occurrence in LMICs. Data around the management of such patients is scarce and leads to variation in practices (15–17). Cyanosis, coagulopathies, diastolic dysfunction, and hyper-viscosity increase post-operative morbidity and mortality in cyanotic heart lesions like the tetralogy of Fallot (7). Similarly, management of critical CHD conditions like transposition of great vessels is remarkably different in LMICs i.e., 2/3rd of patients, in a large global registry of CHD patients from LMICs, presented late and underwent a primary arterial switch operation beyond 4 weeks of age while 20% cases had a two-staged arterial switch (18).

### How Much Is the Data Deficit?

Lack of contextually applicable guidelines leads to the use of HIC guidelines which can be impractical or even impossible and may lead to heterogeneity in care pathways and variability in outcomes (19, 20). LMICs account for 90% of the world's CHD population, yet there is an extreme dearth of data and health care research originating from these countries. The Global Burden of Disease 2017 study on CHD reports little to no direct data on CHD outcomes from a majority of the LMICs (4).

## Barriers to Evidence Generation in LMICS

Despite realization of the importance of contextual research and evidence generation for local, cost-effective solutions in the LMIC, progress has been sluggish and uncoordinated. Therefore, it is imperative to recognize the barriers to conducting research and availability of data in LMICs so that systemic solutions can be designed to address them. These barriers can be broadly classified as following:

### Competing Public Health Priorities Leading to Inadequate Funding and Resources for Complex Care CHD Research

External funding comprising international donors, grants, and research collaborators encompass 90% of the resources for research in LMICs (21). However, the primary agenda for the largest funding health agencies in LMICs like the USAID, UKAID, Welcome Trust, WHO, UNICEF, Bill and Melinda Gates Foundation, and The Global Fund, continues to be infectious diseases, vaccines, and nutritional disorders. These diseases remain the focus in global health like Sustainable Development Goals indicators, driven mainly by donor interest. From 1990 to 2015, in LMICs, infant mortality rates dropped by more than 50% for infectious diseases and protein malnutrition while the death rate from CHD persisted, springing it up to the 5th leading cause of death (3). Despite this changing landscape of disease spectrum, minimum funds are allocated for evidence generation and research around CHD in LMICs. Policy Cures Research, a global health body for research and data collection and analysis, reported that 70% of the total neglected disease investment focused on HIV/AIDS, tuberculosis, and malaria in 2018, while CHD did not fall in their neglected diseases panel, and acute rheumatic fever received only 0.1% of this funding (22). Deep-seated disparities in the process of fund allocation are further highlighted by a survey conducted in 2010 where 85% of grant reviewers felt inadequately trained in grant review (23).

### Lack of a Culture That Values and Supports Research

Many academic medical centers or health care delivery institutes in LMICs fail to adequately recognize their role in developing a clear vision around contextual evidence generation and research to identify more cost-effective measures of health care delivery. Frequently health care “leaders” in LMICs are unable to understand that contextual data and research is probably the most effective way of improving the health care outcomes in their countries (19). Apart from a few large tertiary care centers in LMICs, most local organizations do not invest in or support research conferences, workshops, or reward research productivity as a part of their academic objectives (24). Centers also provide more financial incentives for extra clinical productivity rather than for research work, thus making research work a lesser priority. Moreover, LMICs have a lower patient to physician ratios resulting in competing clinical demands, thus leaving inadequate time for quality research for the inundated physicians working in academic settings. For example, CHD prevalence in Pakistan is comparable to the United States, yet compared to the US there are < 5% pediatric cardiac care centers and < 1% of the pediatric cardiologists present in the country to manage overwhelming burden of disease (17, 25). Similar trends are seen in many other LMICs i.e., 0.04 adult cardiac surgeons and 0.03 pediatric cardiac surgeons per million population, compared with 7.15 adult cardiac surgeons and 1.67 pediatric cardiac surgeons in high-income countries (26). There is a lack of tenured or ring-fenced positions in institutions to incentivize the return of those clinician-scientists who have acquired post-graduate research outside their countries (27).

### Limited Capacity to Do Research or Generate Evidence

While the challenge of resources and funds can be addressed through different means (e.g., philanthropy), mentoring local researchers to pursue careers in clinical research is another tremendous challenge. These challenges are mainly due to a scarcity of infrastructure with very few research laboratories, proper equipment, organized national databases and skilled human resources (analysts, masters level, and Ph.D.- level graduates, statisticians, data scientists, etc.) (28). A lack of vision and incentivization to generate local evidence also transcribes into limited mentorship of young physicians and emerging health leaders in the importance of local data and contextual research. Additionally, weak governance and accountability around mentorship lead to inconsistent and ineffective coaching of the early career physicians. Few health care providers with limited research skills enter the workforce not adding much to the national capacity to do high quality research or evidence generation (24).

### Poor Representation in Global Health Forums

Poor representation of researchers from LMICs (29), a hierarchal relation between HIC and LMIC research collaborators (30) and a significant publication bias (31) against data from LMIC points toward a disparity between the research/researchers from the HICs vs. those in LMICs especially within the arena of global health (32). Such a disparity is yet another reason for lack of evidence from LMICs.

### Poor Representation of LMIC Researchers

Global health forums address issues of insufficient resources, funding, and capacity in LMICs. However, for over a decade the Global South (predominantly LMICs) continue to receive sparse representation on leading global health boards and executive councils. It is estimated that 85% of the global health organization headquarters are in the Global North (29). These organizations have established advocacy pathways and funding resources to address global health problems in the Global South which may not be contextually relevant (29). The Consortium of Universities for Global Health (CUGH) is one of the world's largest academic-based organizationss established in 2007 to support academic institutions in addressing global health challenges. In 2016, only two out of the 16-member Board of Directors of CUGH were from the Global South (31). This inequity steers most of the capacity building and training for academic programs in global health toward the Global North while the disease burden exists in the South. Similarly, commissioners from HICs led 72% of the Global Surgery and 73% of the Global Health Lancet commissions (31). Due to the massive underrepresentation of LMIC attendees in global health conferences, the issues facing them are not brought to the forefront. Barely 4% of academic conferences are held in low-income countries, representing <40% of delegates from LMICs (29). This poor representation is due to financial constraints, visa restrictions, political barriers, and failure to actively seek and identify expertise from LMICs (29). Even amongst the low numbers of attendees from LMICs, those that attend have minimal active speaking opportunities, research presentations, and support, thus making their voices left unheard (29). When clinicians and researchers have no opportunities to participate in decision-making panels, it hampers their research interests as their efforts seem to add no significant value to impact clinical guidelines.

### Hierarchal Relation Between HIC and LMIC Research Collaborators

In research collaborations between HIC and LMICs, when decisions and conversations partake, the voices and concerns of these researchers in LMIC (usually taking role of “gatekeepers)” remain typically absent. As a result, plans made through such “collaborations” do not align with the academic programs, timetables and teaching systems of centers in the south. This uni-directional “gatekeeping” attitude to “facilitate” scholars from the Global North consumes more resources, time, energy, with little to no emphasis on improvement opportunities (30). An additional challenge is that funding is directed usually toward institutions in the Global North and researchers in Global South being dependent on the funds tend to not speak about these issues and may continue to suffer in silence. The dependence on such funds is also a reason for institutions in LMICs to not support their researchers when they share their concern about such hierarchy in collaborations (33, 34).

### Publication Bias

Publications in reputable global health journals allows dissemination of identified local challenges and contextual sustainable solutions and thus can direct future policymaking. The power imbalances within global surgery academia are at risk of being transferred to national policy and adversely affecting resource allocation (35). In an ideal situation, even if LMIC researchers tackle all the major gaps in resources and representation to conduct locally relevant research, publication bias still limits their credibility and voice. Bibliometric studies of global health publications have reported inequalities in global health research overall, with marginalization and disempowerment of LMIC authors (35). HICs publish almost all global health journals with very few editor-in-chiefs from LMICs and low representation of editors or editorial board members from LMICs (32). Top 12 health journals have 33% LMIC representation amongst editors and editorial board members (31). Amongst 27 specialty global health journals, 68% of editors and 73% of editors-in-chief came from HICs (36). Consequently, there is an increased preference for publishing research conducted by the developed countries of the Global North (31). This disparity was further aggravated amongst leading female roles from LMICs, encompassing only 4% of leading female editors belonging from LMICs amongst the top 12 health journals (31). LMIC female first authors had manuscripts published in journals with impact factors ~ 14 points lower than papers from their HIC counterparts (36). A systematic analysis of current trends in authorship demographics for global surgery publications reported 51% of authors affiliated only with HICs with over two-thirds of first and last authors were affiliated with at least one HIC institution (35). Elite journals frequently reject papers from LMICs with the presumption of it being of questionable data quality, not of interest to their reader or findings not being generalizable to HICs. Subsequently, authors are advised to submit their articles to local low-impact journals resulting in lower reach and hence limited citations. Unaffordable publication costs of other impactful global health journals add to this engrained disparity and further restrict southern data from being published in reputable journals (37).

## Solutions–The Way Forward

**Figure 1:**
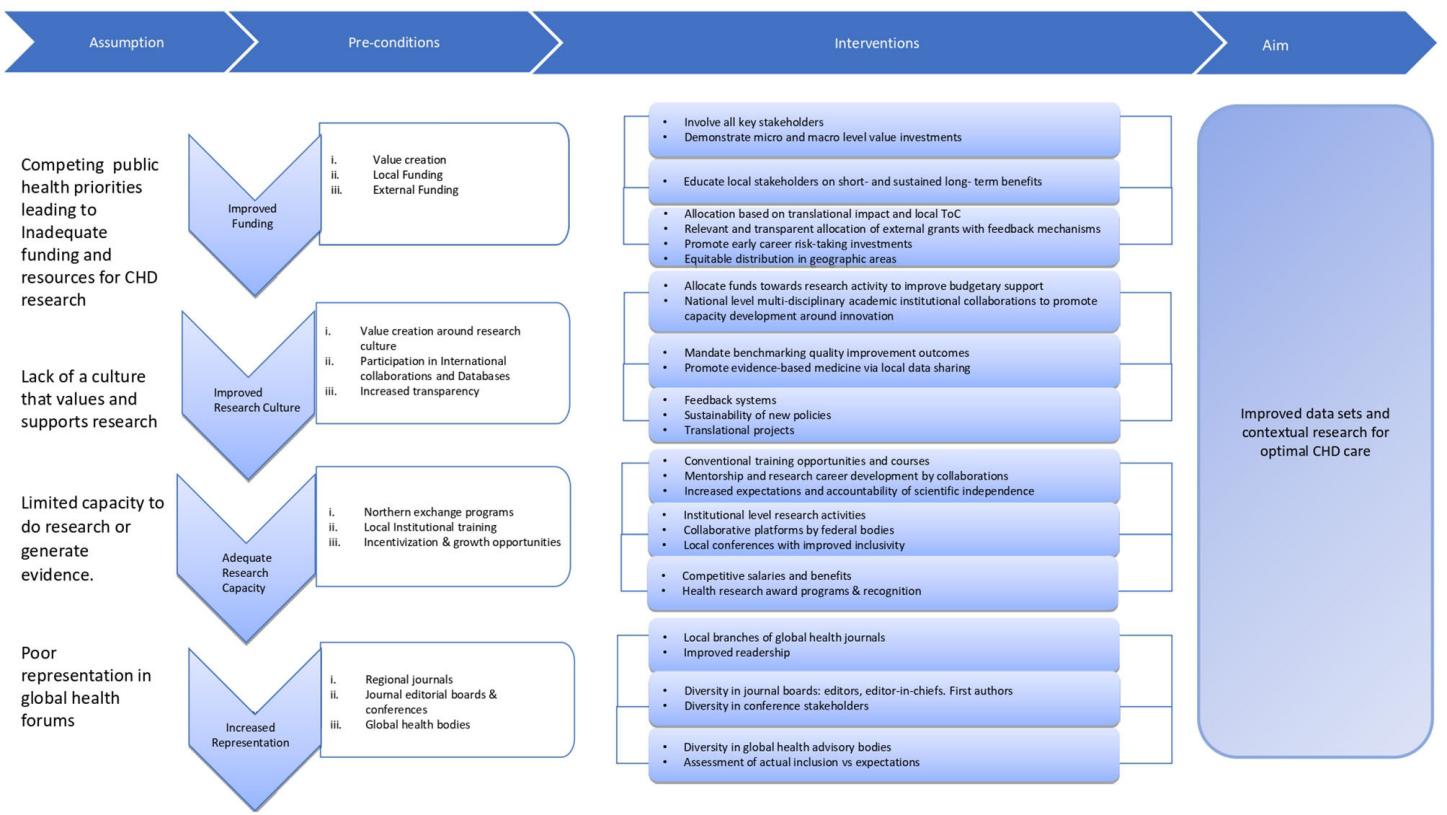
Theory of change around increasing data generation and contextual research output in LMICs.

The most essential aspect of improving research in LMIC is the realization and ownership of the problem around paucity of local evidence by patients, health care providers, academic centers and governments in these countries. In 2015 alone, poor quality health care in LMIC led to ~ USD six trillion in economic losses (38). Thus, all stakeholders must understand that valuing and investing into data/evidence generation is imperative for their own benefit. Nationally tailored plans increase ownership of problems and even though they might not be perfect, they are likely to have a greater impact (19). Moreover, Southern voices can only demand inclusivity in global health forums if they strengthen their systems, collaborate, come forward as a unified representation to add value to global data. Thus, LMICs must identify the challenges (as described above) and create a clear theory of change (ToC) to address these challenges at a micro- (individual patient or physician or institutions delivering health care) and a macro- (government and health ministries) level. ToC has been widely used and it is considered to be the basis of monitoring and (39) theory-based evaluations (40–42). According to Weiss, a ToC is, “a theory of how and why an initiative works (43).” A robust ToC includes several components that makes the model more systematic (39) i.e., preconditions, interventions, outcome and goal. Preconditions are a necessary requirement, condition or element that should be present for achieving the desired outcome while to fulfill these preconditions it is important to have efficient interventions that helps achieve the outcomes and impact of interest (39). Such ToCs are complex and need a very thoughtful approach. We propose a ToC, its pre-conditions, interventions and metrics (process and outcome) to measure the effectiveness of the intervention to enhance research output from LMICs (Figure 1, Table 1).

**Table 1:** A theory of change model to increase data generation and contextual research in low- to middle- income countries.

**Impact:** Improved evidence generation and contextual research for optimal CHD care ***N*** (%): Increase in available health outcome metric datasets, increase in number of papers on data from LMIC authored by investigators from Global south.		
**Intermediate Outcome:** Improved resources, representation, and publications ***N*** (%): 1. Increase in the number of resources: Amount in $ funding toward health outcome research in LMIC and number of high quality LMIC researchers and other supporting staff (e.g., statisticians, data scientist etc) 2. Increase diversity in global health advisory, editorial and conference representation 3. Increase in the number of publications and citations from LMICs lead investigators.		
**Pre-conditions**	**Interventions**	**Indicator/Metrics**
**1. Improved Funding**		
1.1 Value creation around contextual evidence and research among local stakeholders	• Involving key stakeholders (patients, physicians, insurance companies, pharmaceuticals/device companies, government health ministries). • Demonstrating value in investing in data especially health outcome metrics at a micro (individual- physician or patient or institutional- hospital, academic medical centers) or a macro-level (government- health ministries)	– Number of disease specific health outcome registries or collaboratives created nationally. – Number of block chain start-ups managing health outcome data emerging locally. – Number of national conferences or meeting around contextual data generation and health outcome research including all stakeholders.
1.2 Increased local funding	• Creating holistic impact outcomes for healthcare workers and institutions thus tying in health outcome data and research to improved human capacity and productivity i.e., a healthy individual will have less working days lost and less expenditure on a healthy workforce for company with health insurance benefits. • Demonstrating benefits of value-based health care to pharmaceuticals/device industry, insurance companies. • Incentive for government health authorities to invest in data generation and research around health outcomes thus helping them objectively and effectively allocate health budget. • Educate stakeholders (industry, insurances, government, philanthropist etc) about better return of their investment through data and research driven improved quality of care.	– Number of health care providing entities collecting and providing health outcome data. – Number of corporate health care entities (device companies, pharmaceuticals) with dedicated health outcome funding budgets.
1.3 Contextually relevant external funding	• Increased transparency of external grants by ensuring allocation of external grants alignment with the national health care needs and in consultation with the community. • Prevent centralization of resources by funding research in all geographic areas regardless of economy status	– Amount (in $) of research funds allocated by health care philanthropic organizations, government and insurance companies – Reports by large funding agencies of N(%) of local stakeholders involved in grant evaluation. – Amount of (in $) external funds allocated to the disease areas causing the highest morbidity and mortality. – Geographic mapping of percentage of funding by large funding agencies – Amount (in $) amount allocated to health care facilities clearly demonstrating holistic impact outcomes.
**2. Improved research culture**		
2.1 Value creation around institutions promoting contextual research and capacity building	• Preferential health budget support of federal funds to institutions demonstrating robust research activities, improvement in healthcare based on contextual data generation and research capacity building. • National academic institutional collaborations amongst various schools–health, humanities, business, engineering, information and technology–to promote a culture of innovation and entrepreneurship to tackle healthcare challenges using a multi-disciplinary approach. Technology transfer and opportunity to create health care startups and revenue generating opportunities. • Acknowledging institution which has employee appraisal based on their contextual research productivity. Encourage institutions to value impact on health outcome vs. journal impact factors or number of publications.	– N (%) of clinicians and researchers at government and private health institutes involved in conducting research – Number of scalable technologies and startups created yearly from graduates of such academic institutions. – $ funding to institution fulfilling the criteria of a “research” valuing center.
2.2 Increased participation in international database and collaboratives.	• Mandate (at a national level) benchmarking of outcomes and quality improvement sciences. Highlight programs with improved outcomes as determined by a third-party auditor (i.e., IQIC) • Encourage local stakeholders within multiple LMICs to collaboratively propose optimal and cost-effective solutions to prevalent problems by sharing their experiences and promote the practice of evidence-based medicine.	– Number of health care delivery entities benchmarking their health outcomes by being on national or international registries.
2.3 Increased transparency around funded research outputs and health outcome data	• Encourage focus on knowledge translation and innovative projects pertaining to local solutions • Develop feedback systems to monitor alignment of resource allocation and outcome improvement. Monitor newly introduced modified policies to ensure sustainability.	– Number of nationally funded projects leading to contextual clinical practice guidelines – Number of nationally funded research projects demonstrating improvement in health outcomes.
**3. Adequate capacity to do conduct research activities**		
3.1 Improved training *via* northern exchange programs and mentorship	• Online and in person certificate training programs and workshops of conventional research courses to target larger masses. Sponsored master's and doctorate degrees, postdoctoral research positions to LMIC grantees and trainees. • Teaching translational and implementational science to encourage innovation and contextually relevant research that may improve practice and policy • Assign mentors to these trainees that can give detailed feedback and offer continued assistance throughout their journey promoting research career development. Sustained mentorship should be lauded, supported and programs demonstrating it prioritized in receiving global health funds. • Increased expectations and accountability from exchange program awardees to build research developmental programs at home institutes and achieve long term scientific independence.	– Number (%) of exchange programs awardees from LMICs every year at HIC global health institutes. – Number (%) conference presentations and manuscripts led by LMICs mentees – Number (%) of research programs developed by LMICs mentees in their setups
3.2 Training provided by academic institutions within LMICs	• Institutional level journal clubs and seminars encouraging lower resourced health care setups to participate, voice and formulate research agendas. • Collaborative platforms by federal bodies to address problems and suggest sustainable solutions through modified interventions. Modified care practices need to be documented initially at a local level *via* publications in local journals with a larger goal of establishing national database and guidelines to support these claims. • At a national level, encourage local health care workers with limited resources and large patient loads to exchange ideas and information at health conferences as participants, panelists, speakers, and advocates to bring neglected local health problems to the table. Promote large scale evidence-based medicine practices by defining clear outcomes and objectives of such forums.	– Number (%) of research related activities conducted every year at leading academic health institutes. – Number (%) local specialized journal publications and their quality of work. – Number (%) of national health conferences and participants diversity across the country.
3.3 Incentives and fair growth opportunities	• Competitive salaries and benefits for health care workers conducting research in the communities at government and private institutional levels. • Providing technological expertise and tools for improved data acquisition and health information exchange. • Health research award programs need to acknowledge high quality research work aimed at reporting large data, quality improvement initiatives, creating contextual guidelines and demonstrating improved health outcomes.	– Number (%) of national awards given to researchers to recognize their efforts in improving health outcomes. – % increment in salaries of researchers based on their contribution toward improving health outcomes nationally.
**4. Increase representation in global health journal, conferences, and governance bodies**.		
4.1 Regional journals to target relevant audience.	• Encourage separate local or regional branches of global health journals which may publish more relevant and applicable data from local researchers. • Encourage utilization of publication and the importance of quality of work over journal impact-factor.	– Number of leading global health journals having subsets of regional journals.
4.2 Diversity amongst journal editorial boards and conferences.	• Increase diversity of representation amongst all journals and conference stakeholders. This may apply to editor-in-chiefs, editors, first authors of published articles for top global health journals. This may also extend to speakers and participants at global health conferences.	– A diversity score similar to the Composite Editorial Board Diversity (CEBD) score reported by [Bhaumik and Jagnoor (44)] can assess geographic, ethnic, and country income-level diversity.
4.3 Inclusion amongst global health bodies	• Systematic reporting system to keep track inclusion in global health advocacy bodies • Liaisons with researchers active in eliminating disparity of representation.	– Yearly audits of number (%) of LMIC representation in advisory bodies and editorial boards.

### Pre-conditions and Interventions of the Theory of Change

#### Improved Funding

**Figure 2:**
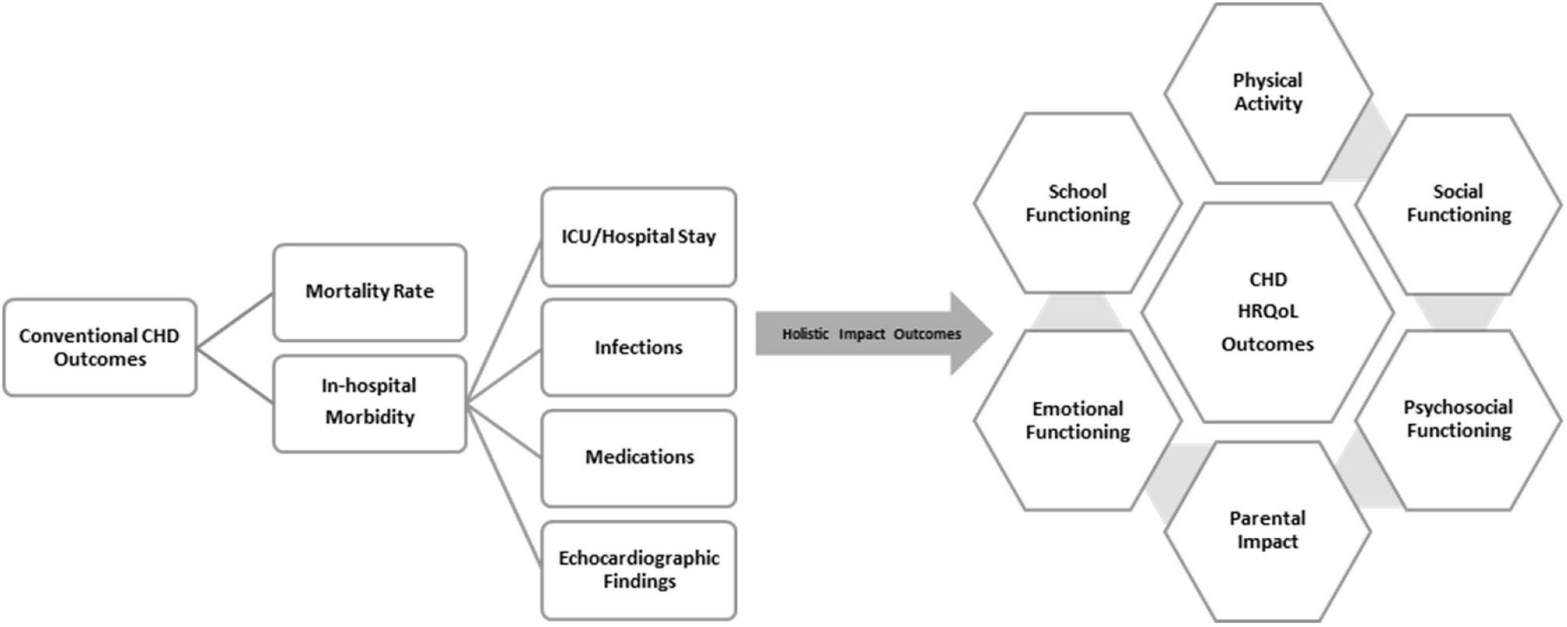
Conventional vs. Holistic health outcomes in CHD; HRQoL, Health related quality of life.

It is essential that funds are available to generate evidence and conduct contextually relevant research. Without funds, any effort toward creating value around evidence generation and research activities is not sustainable. The value of having high quality holistic outcome data and contextual research especially around quality improvement must be recognized by all stakeholders i.e., patients, health care providers, health care delivery facilities and institutions, insurance companies, health care industry and health ministries/government. Holistic health outcome data (encompassing the patients mental, physical and social wellbeing) (Figure 2), contextual research around service delivery (to improve attainment of healthy state, degree of health recovery and sustainability of health- the three tier of value-based health care outcomes) and innovation can benefit the health care ecosystem at a micro- (individual and institutional stakeholders) and a macro-level (government and health ministries). If and when this buy-in from local stakeholders comes, funds provided by them will be more readily and sustainably available. This realization among stakeholders and subsequent value around contextual evidence generation and research can be created through several ways:

##### Value Creation Around Data and Research Among Local Stakeholders

It is a win-win situation where all the stakeholders can benefit if they invest in local evidence generation and contextual research.

**Patient/Family Unit as a Stakeholder**. Access to data will give patients the autonomy to select the best value for their care. Patients can save more money as poor quality of care is more costly and also leads to more mistrust in the health system which makes them doctor “shop” (38).

**Physician and Health Care Delivery Institution**. Ability to measure and share their outcomes can give these stakeholders an opportunity to increase their marketability of service, referral, and remuneration from insurance companies, pharmaceutical companies, and the healthcare industry. For example, in Pakistan, atherosclerotic cardiovascular disease (ASCVD) is one of the leading causes of early mortality in young adults with no subsequent assessment of risk factors within the population. The first-ever largest longitudinal cohort study in Pakistan, PAK SEHAT, is being implemented in collaboration with a specialized heart hospital, Tabba Heart Institute and Getz Pharma (https://www.dawn.com/news/1659222) to formulate a comprehensive database on the native Pakistani population to assess the likelihood of developing ASCVD. This serves as a great example of local stakeholders contributing towards locally relevant research in ways that can also be beneficial for them.

**Insurance Companies, Industry, and health care philanthropists**. In many LMICs, the majority of the cost reimbursement is out of pocket payments (45). Limited health insurance, government subsidies and philanthropists bear the cost of care for patients who cannot afford treatment (the overwhelming majority in LMIC) (46). Having access to health outcome data and quality improvement research may be of interest to these stakeholders as it gives them an opportunity to effectively use their funds. Health care startups like block chain will also find space in such an environment and becomes another stream of value creation and incentives for people to invest in local research activities.

**Government**. Access to health outcome data and local research can be of value to governments. It helps them achieve good health indicator-based international ratings, which helps governments in LMICs procure more funds and aids from bodies like the International Monetary Fund (IMF) or the World Bank. It also helps them in effective and objective use of their health budgets. Increased medical tourism is another incentive for governments in LMIC to invest in contextual data generation and research. These incentives can make the stakeholders value data and research and thus invest in it as they see it as something that will give them a good return on their investment (ROI).

##### Increased Local Funding

Demonstrating “ROI” as money saved by improving health outcomes through local innovations and contextually relevant management may help in more sustained local investment. A dialogue with all the stakeholders along these lines will help direct their focus on short- and long-term benefits of improved quality of care offered by investing in contextual research and data generation.

##### Contextually Relevant External Funding

Research funding by external bodies should be allocated based on translational impact and according to the local ToC (47). This has to be mandated by governments in LMICs trickling down to academic and research organizations as this is in the best interest of their country's health needs. External funders will have to respect the needs of the local communities and must realize that their “best” intentions may not necessarily be in the “best” interest of the local communities. One way of assessing these needs would be to involve the local health care stakeholders in the grant evaluation and monitoring process. Feedback mechanisms should also be encouraged to assess long-term progress amongst funded projects and whether the funding indeed has impacted disease burden and local challenges. Transparency of the grant evaluation process needs immediate attention (23) and the concept of repeatedly funding the “safe” researcher (48, 49) needs to be abolished. Building local capacity will only happen by encouraging early career researchers by funding their work even if they seem to be a “risky investment.” Evaluation of investigators must move away from just publications to actual impact. The San Francisco Declaration on Research Assessment (DORA) points out that using the Journal Impact Factor as a proxy measure for the value or quality of specific research may be erroneous and one-dimensional and lead to a biased research assessment and perpetuates the emphasis on publication in certain high-tier journals without a more comprehensive assessment of impact. Another strategy would be to prevent centralization of resource allocation and promote equitable distribution in all geographic areas (50).

### Improved Research Culture

Waiting to develop a culture of research only once adequate resources are available, may never fill the research gaps. The approach should be to foster a culture of innovation that can encourage research activities within the available resources. A few successful innovations can subsequently drive more funds. This can be done through several different ways.

#### Value Creation Around Institutions Promoting Contextual Research and Capacity Building

Academic medical centers should be encouraged to engage in data generation and research and should be incentivized by giving such centers better budgetary support. Private centers should allocate funds toward research activity, publication costs etc. for clinical research mandated by the government. A government-level oversight will ensure appropriate utilizations of the funds by these centers. Other institutions (business schools, health care leader mentorship programs etc.) should educate their students on the value of data and its objectivity and create data-centric systems to aid in decision-making. They can further educate other stakeholders in the society to propagate a culture of research and data-driven health care. Creating national level collaborations between engineering, business, social science and health care institutions will help develop capacity around innovative fields like data science, artificial intelligence, social innovation and entrepreneurship.

#### Increased Participation in International Databases and Collaboratives

Participating in international databases and collaboratives is a good start to maintain data and build local biorepositories. The International Quality Improvement Collaborative (IQIC) for CHD surgery and catheterization, a web-based platform providing benchmark data on clinical outcomes and resource utilization metrics, is an exemplary template for the collaborations that need to be developed. IQIC allows all institutions, lacking a national database or sufficient resources, to benchmark outcomes and compare their analyses with other LMICs centers. In addition, it also encourages collaborative learning through free webinars and supports local efforts in research activities and manuscript writings. Such measures will allow routine interaction amongst global institutions to share practices and care protocols. It will facilitate conversations between participating hospitals with similar resource backgrounds, who desire to improve the quality of their care and optimize cost-effectiveness and share their experiences through publications (51).

#### Increased Transparency Around Funded Research Outputs and Health Outcome Data

Feedback systems with quarterly reports to assess the progress of research developments can monitor resource allocation and outcome improvement. Meanwhile, newly introduced evidence-based modified policies will have to be monitored regularly to ensure that change sought *via* these frameworks remains sustainable. Nevertheless, emphasis should be on translation projects pertaining to local issues rather than replicating work done in HICs in a controlled, artificial setup that often cannot be replicated.

### Adequate Capacity to Conduct Research Activities

#### Improved Training via Northern Exchange Programs and Mentorship

Individuals or experts working in the global health arena should be encouraged to act as mentors to early career researchers or clinicians in LMICs. The performance of these experts should be appraised by their affiliated organizations based on the quality of the mentees produced. The long-term expectation should be for these mentees to be autonomous researchers who demonstrate improvement in health outcomes in their respective countries through their work. Such systematic approach of collaborative staff training and knowledge sharing can promote long-term scientific independence (28). A combination of training opportunities such as fellowships, certificate programs, master's and doctorate degrees, postdoctoral positions can be offered to LMIC researchers, especially by programs seeking funding within the global health space. A fixed percentage of grantees and trainees from LMICs should be sponsored to receive these certificate and degree programs with the expectations of building research infrastructures at various institutes in their home countries. While not everyone can be sponsored to train at reputable institutes, utilizing virtual platforms to hold essential research courses at low-to-no costs will allow training in remote areas. Apart from the conventional research courses i.e., research design, grant writing, statistics, a special focus should be given in teaching translational and implementation science so that investigators are primed to be innovative and help catalyze uptake of evidence into policy and practice (52). Use of high quality implementation science has been proposed to understand the contextual factors associated with the scaling of surgical systems in LMICs (53). Such quality evidence around implementation may also help HIC adopt such robustly tested processes in LMICs. These efforts will hone research career development amongst young researchers and improve the workforce. Research collaborations between HIC and LMIC must include capacity building of the LMIC investigators to lead manuscripts and present at various conferences and forums. The accountability of these deliverables has to be monitored by both the collaborating institutions in HIC and LMICs. One initiative in this regard, focusing on growing and disseminating talent in medicine is the Women as One Talent Directory (https://womenasone.org/register/sign-up/) is a robust and sortable online database of talented women in medicine to facilitate professional opportunities and to allow for networking.

#### Training Provided by Academic Institutions Within LMICs

At a micro level, large academic institutes should take the initiative to increase the pool of local researchers. The handful of institutions in LMICs which are adequately resourced should champion the cause and hold mandatory institutional level journal clubs and seminars, which could be linked to units without these remotely. This would promote partnerships with lower resourced health care setups to discuss and formulate contextual research agendas. The performance and subsequent funding of such institutions when linked to improvement in health outcomes, will lead to a more concerted and sustained effort by these institutions to promote a culture of contextually relevant research.

At a macro level, federal bodies should develop collaborative platforms to promote translational research and sharing of resources. These platforms can be utilized to address health inequities and encourage innovative sustainable solutions at the local level. There is a need for the establishment of local health journals where articles addressing community problems and data deficits can be published and disseminated. It will help develop modified interventions targeting the local population at-need and encourage discourse on outcomes of modified care practices. If care protocols are accurately reported and accessible to neighborhood local regions, similar setups can adopt successful strategies, thereby causing a bigger impact. Consistent documentation of these practices in multiple centers enables a wider outreach and acceptance. Furthermore, local health journals should invite small rural health care setups to publish data on their patients. Encouraging data from multiple small centers can help form a larger cumulative database in the future and impactful local guidelines (54). The student workforce at teaching hospitals can be given opportunities to participate in data collection to set up these large databases or do mini-research projects. They can use incentives of developing their resume and research mentorship from faculty to participate in these studies.

National governance bodies need to establish health conferences at a national level as an accessible medium for exchanging ideas and information. Such conferences need to have clear objectives and defined outcomes (i.e., establishing of local databases, exchanging ideas, creating research consortiums, tracking progress in heath outcome metrics, etc.). Such a focused approach will help reap the maximum benefit of such gatherings as such conferences are costly and require intense organizing efforts. Health care workers with limited access and large patient loads should be given special attention. They should be encouraged to participate as panelists, speakers, and advocates. It will encourage local stakeholders to gather data through mutual collaboration which can result in comprehensive solutions to prevalent problems and promote the practice of evidence-based medicine.

Once local partnerships are forged and local centers are driven toward establishing national healthcare policies through research and development, partnerships with programs in other LMICs with shared problems and challenges can be considered.

#### Incentivization and Fair Growth Opportunities

In LMICs where financial benefits lie within extra clinical hours, competitive salaries and benefits for contextual research can incentivize most physicians to begin investing their time toward contextual research. Such an initiative should be encouraged at both, government, and private institutional levels. Recognizing efforts and giving due credit through health research awards and national recognition can further enhance their interests.

### Increase Representation in Global Health Journal, Conferences, and Governance Bodies

#### Regional Journals to Target Relevant Audience

While global health journals may find it impossible to be completely inclusive of all regions around the globe, regional subsets of journals may provide more relevant data grouping and improved readership while being cognizant of diversity and inclusivity from all ethnic and income-level backgrounds. This will increase prospects of publication, consolidate ethnic or regional groups of similar data and appeal to a more relevant readership. It will also increase the editorial board members in numbers and provide an incentive toward larger membership and improved diversity on decision making panels. Moreover, an important aspect that stakeholders (e.g., academic institutions) need to consider is the utilization of the publication. Value has to be placed on the quality of work and its significance toward achieving the outcomes rather than the impact-factor of the journal it gets into (37).

#### Diversity Amongst Journal Editorial Boards and Conferences

Global health journals can support reduction of data disparity by ensuring greater diversity of their editorial boards ranging from directors to editors to promote a balanced and fair perspective. Such an effort can provide a larger number of peer-reviewers and encourage submission from studies conducted in more diverse backgrounds, thus providing a better global health perspective. A commitment for blinded reviews from high-tier journals would be welcomed to preserve the quality and integrity of research reviews (55). Composite Editorial Board Diversity Score (CEBDS) (44) evaluated the diversity in top 27 global health journals amongst gender, geographic and income level regions serving as a great example to quantify actual diversity. A detailed assessment based on a globally approved scoring system may help target the gaps in inclusivity amongst editorial bodies. Furthermore, an assessment of first authors of all publications and their citations can help reflect upon the diversity of acceptance from research conducted in different backgrounds. The data can also be used by institutions in LMIC to advocate for more equitable partnerships. Thus, more inclusion will allow addressing problems in acquiring data at a global level. It will help devise global initiatives to assist in building capacity and funding projects to collect data from LMIC.

#### Inclusion Amongst Global Health Bodies

A goal for the pediatric heart disease researchers should be to have a unified voice in global forums like the Sustainable Developmental Goals (SDGs) to ensure the disease remains a national priority. This makes the case significantly easier with governments. Moreover, global health advocacy boards and global health journals should also work to increase representation from LMICs through ensuring accountability. Devising a systematic reporting system to keep track of “expected” vs. “real” inclusion of LMICs in global health will be fruitful. Measures should include mandatory yearly audits, reporting the percentage of LMIC representation in advisory bodies and editorial boards. These yearly audits will give concrete numbers to point to the reality of inclusivity and depict trends of improvement overtime. Liaison with groups of researchers active in the space of addressing the disparity in representation in global health will help magnify the efforts toward meaningful inclusivity of LMIC stakeholders in global health (56).

## Conclusion

There are significant global inequities amongst the division of global health burden of pediatric cardiac disease and allocation of resources. These barriers are well known in LMIC countries, but it is essential to enumerate them to set the scene for the much less known systemic solutions to address them. Along with inadequate access to high-quality care, competing health priorities, fragile health systems, and conflicts, there is an increased lag of evidence generation and research from LMICs. Lack of basic epidemiologic understanding of the disease burden in these regions poses a significant knowledge gap as solutions can only be developed and sustained if the scope of the problem is accurately defined. Unaligned funding, poor representation in global health advocacy bodies, limited opportunities to publish in global health journals, and limited opportunities to participate and speak at global health conferences hinder progress. While global health bodies advocate for these problems, they tend to take control over local health challenges without focusing on the necessary investment they require. We have designed the solutions using a 'Theory of Change (ToC)' approach to understand how and ***why*** certain pathways work. As highlighted in the ToCs, the path begins with national ownership at the top leading to empowerment of the LMIC clinician to publish, especially around clinical outcome data. The proposed strategies have been designed in the best interest of all the stakeholders ensuring sustained engagement of the process. LMICs need to take it upon themselves to improve the health of their populations with the intent to gradually decrease dependence on external funds. The realization must be that an external can only help if “we” put in the effort to resolve our complex healthcare challenges. All stakeholders in society from government bodies, health ministries and systems, to frontline healthcare workers and patients need to be invested to address the local health problems. Moreover, interventions can be designed for a more collaborative and effective HIC-LMIC and LMIC-LMIC partnership directed at increasing resources, capacity building and representation for long-term productivity. We believe that given the strength of the ToC model, it can be very well used by HIC centers too. As a next step, the authors will conduct workshops with a group of practitioner clinicians, hospitals, programs, and other relevant stakeholders to refine the ToC and its associated process/outcome metrics. The listed fundamental outcome metrics of each of the strategies in the ToC table will be the desired goal/outcome for the next set of workshops we have with the relevant stakeholders; for example, the first metric is the number of national disease-specific collaboratives or registries created. In our workshop, we will start with this as the outcome and then map the process with the key stakeholders, including physicians, insurance companies, pharmaceuticals/device companies, and government health ministries listed in the second column. The results of these associated process/outcome metrics will need at least 5 years for a measurable change and are beyond this paper's scope.

## Author Contributions

BH: conception and design, formulation of ToC, drafted, critically reviewed, and revised the manuscript. MR: conception and design, critically reviewed, and revised the manuscript specifically the theory of change. AW: formulation of ToC, drafted, reviewed, and revised the manuscript. RK and LZ: conception and design, reviewed and revised the manuscript. All authors contributed to the article and approved the submitted version.

## Conflict of Interest

The authors declare that the research was conducted in the absence of any commercial or financial relationships that could be construed as a potential conflict of interest.

## Publisher's Note

All claims expressed in this article are solely those of the authors and do not necessarily represent those of their affiliated organizations, or those of the publisher, the editors and the reviewers. Any product that may be evaluated in this article, or claim that may be made by its manufacturer, is not guaranteed or endorsed by the publisher.
